# Supplementary material for: Inhibition of Wnt/β-Catenin Signaling Sensitizes Esophageal Cancer Cells to Chemoradiotherapy
Source: Int J Mol Sci. 2021 Sep 24;22(19):10301. doi: 10.3390/ijms221910301 (PMC8509072; doi:10.3390/ijms221910301)
Supplement: Supplementary file 1 [file ijms-22-10301-s001.zip › ijms-1381041-supplementary.pdf]

International Journal of *Molecular Sciences*

MDPI

Supplementary Materials

Inhibition of Wnt/β-Catenin Signaling Sensitizes Esophageal Cancer Cells to Chemoradiotherapy

Melanie Spitzner [1], Georg Emons [1], Karl Burkhard Schütz [1,2], Hendrik A. Wolff [3,4], Stefan Rieken [3], B. Michael Ghadimi [1], Günter Schneider [1] and Marian Grade [1,*]

Supplementary Figures and Figure Legends

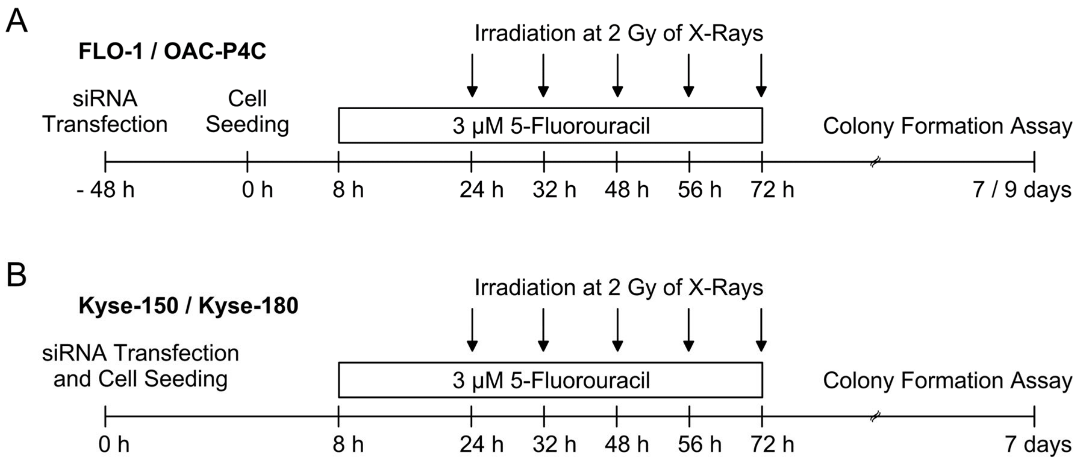

Figure S1. Experimental setup of fractionated irradiation experiments in esophageal cancer cells. (**A**) FLO-1 and OAC-P4C; (**B**) Kyse-150 and Kyse-180.

Figure 1A

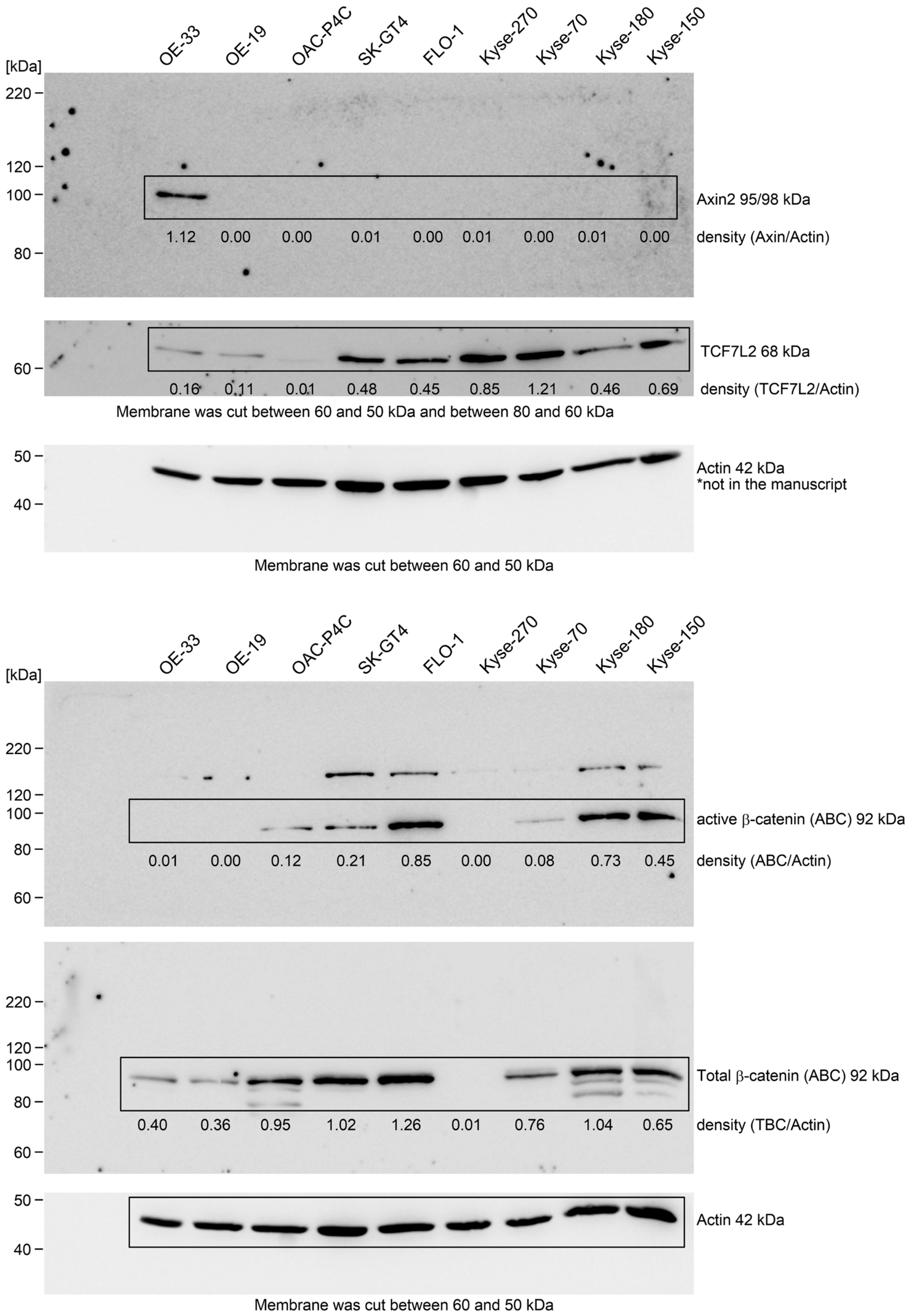
OE-33
OE-19
OAC-P4C
SK-GT4
FLO-1
Kyse-270
Kyse-70
Kyse-180
Kyse-150
[kDa]
220
120
100
80
Axin2 95/98 kDa
1.12 0.00 0.00 0.01 0.00 0.01 0.00 0.01 0.00
density (Axin/Actin)
60
TCF7L2 68 kDa
0.16 0.11 0.01 0.48 0.45 0.85 1.21 0.46 0.69
density (TCF7L2/Actin)
Membrane was cut between 60 and 50 kDa and between 80 and 60 kDa
50
40
Actin 42 kDa
*not in the manuscript
Membrane was cut between 60 and 50 kDa
OE-33
OE-19
OAC-P4C
SK-GT4
FLO-1
Kyse-270
Kyse-70
Kyse-180
Kyse-150
[kDa]
220
120
100
80
60
active β-catenin (ABC) 92 kDa
0.01 0.00 0.12 0.21 0.85 0.00 0.08 0.73 0.45
density (ABC/Actin)
220
120
100
80
60
Total β-catenin (ABC) 92 kDa
0.40 0.36 0.95 1.02 1.26 0.01 0.76 1.04 0.65
density (TBC/Actin)
50
40
Actin 42 kDa
Membrane was cut between 60 and 50 kDa

Figure 2A

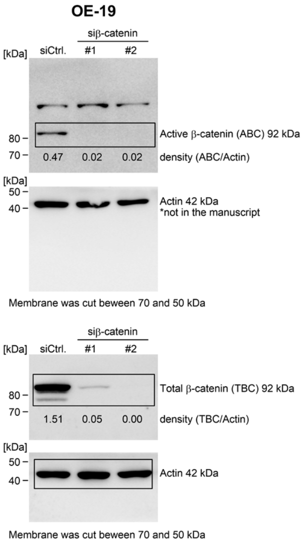

Figure 2B

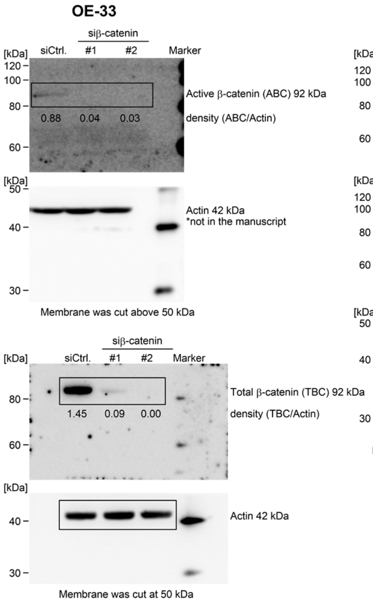

Figure 2C

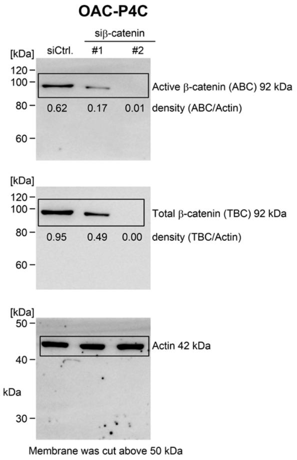

Figure 2D

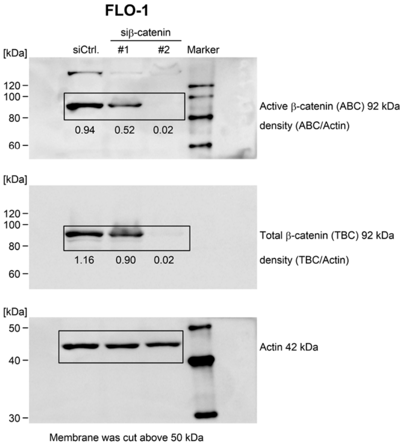

Figure 2E

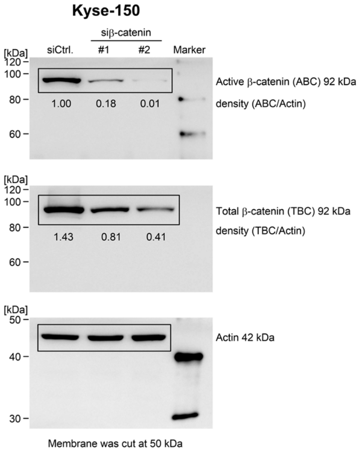

Figure 2F

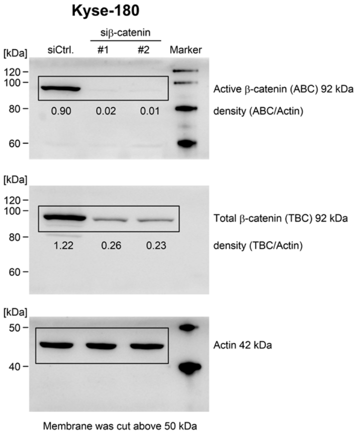

Figure 3A

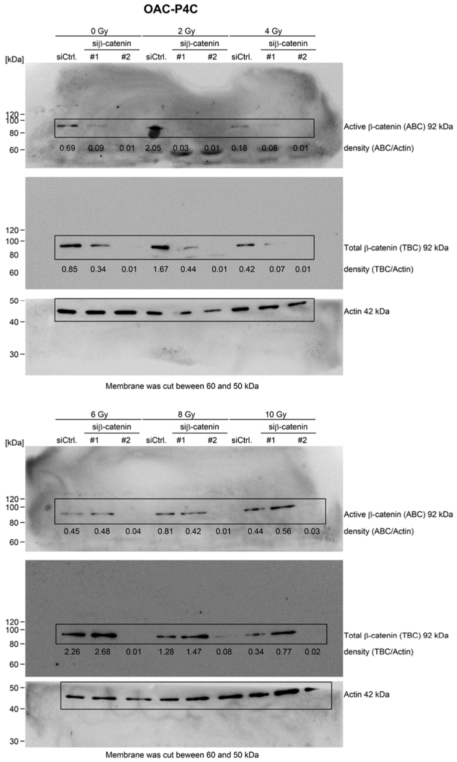

Figure 3B

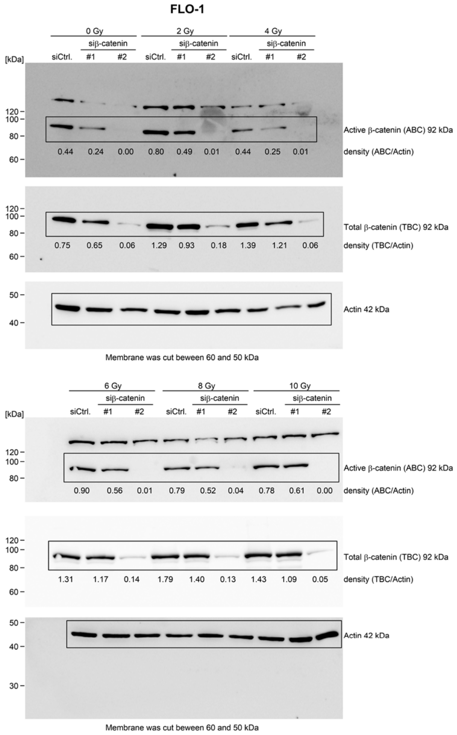

Figure 3C

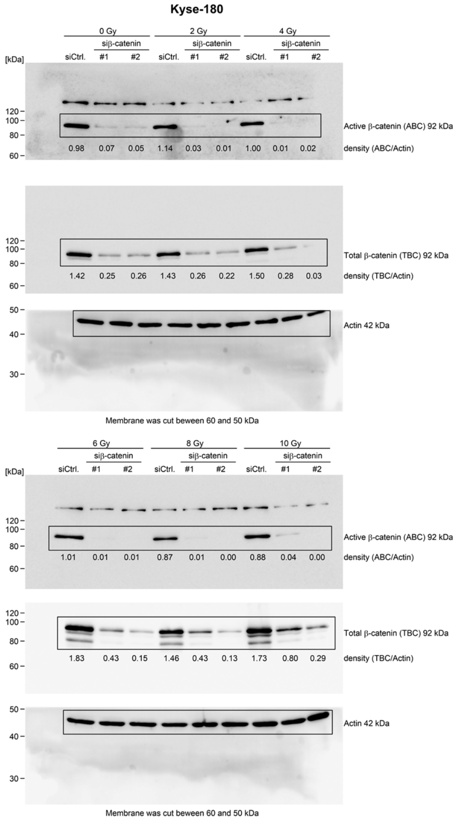

Figure 3D

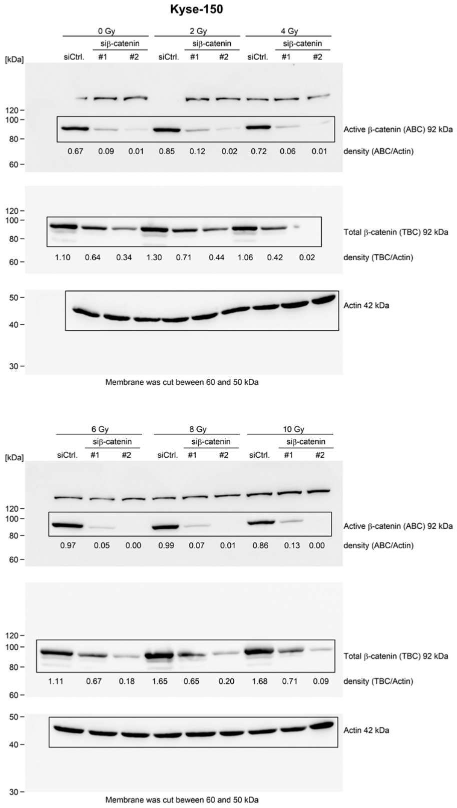

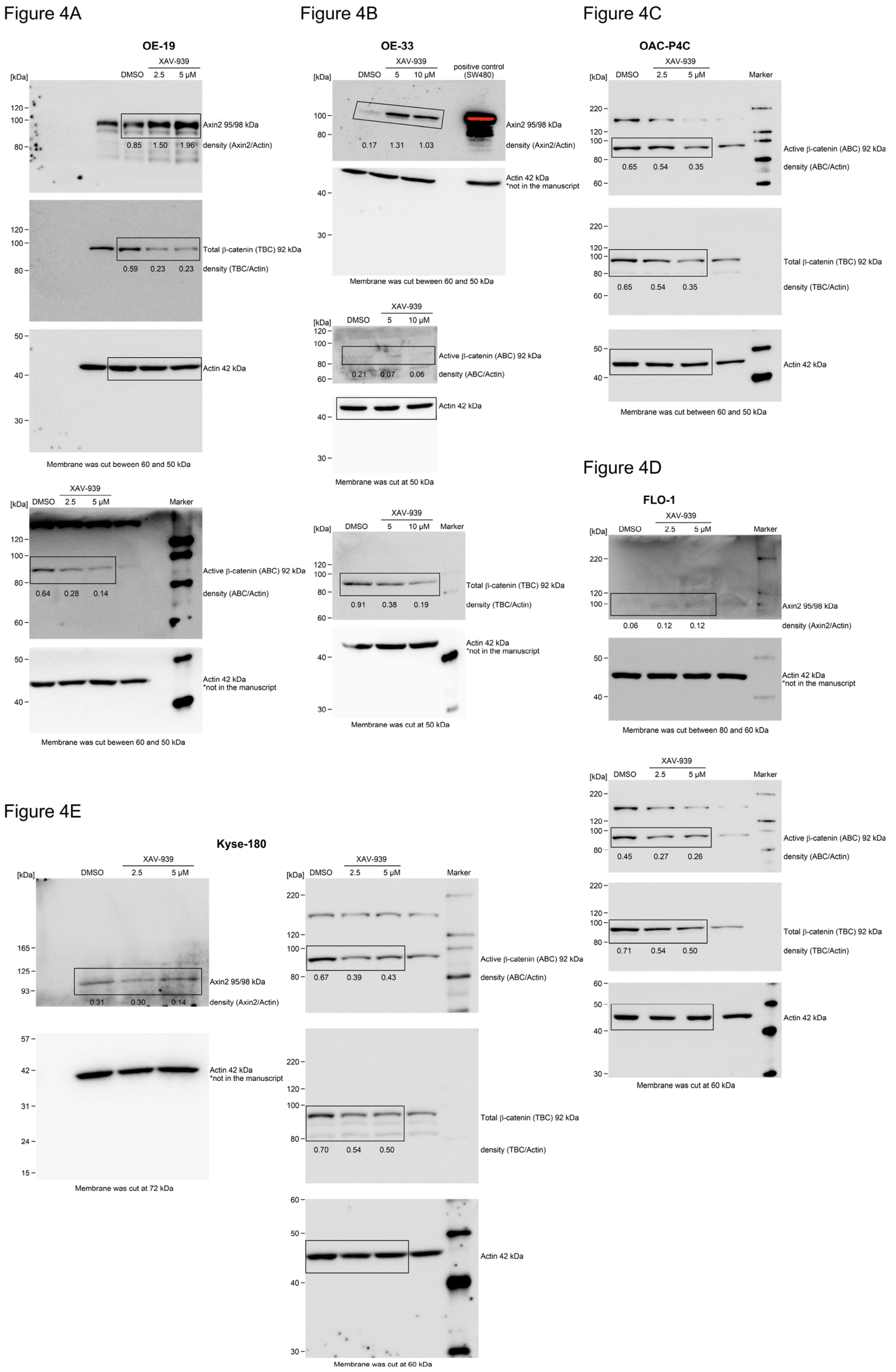

Figure S2. Original immunoblotting images presented in the paper.

Supplementary Tables

Table S1. *P*-values of irradiation experiments and viability assays.

Cell line	Treatment	*P*-value CRT[1]	*P*-value CTB[2]
OE-19	RT vs. CRT at 6 Gy	0.0049 (Fig. 1H)[2]	n.a.
OE-33	RT vs. CRT at 6 Gy	0.0468 (Fig. 1H)[2]	n.a.
OAC-P4C	RT vs. CRT at 6 Gy	0.5218 (Fig. 1H)[2]	n.a.
SK-GT-4	RT vs. CRT at 6 Gy	0.4926 (Fig. 1H)[2]	n.a.
FLO-1	RT vs. CRT at 6 Gy	0.3760 (Fig. 1H)[2]	n.a.
Kyse-70	RT vs. CRT at 6 Gy	0.3412 (Fig. 1I)[2]	n.a.
Kyse-150	RT vs. CRT at 6 Gy	0.6709 (Fig. 1I)[2]	n.a.
Kyse-180	RT vs. CRT at 6 Gy	0.0320 (Fig. 1I)[2]	n.a.
OE-19	siCtrl. vs. siβ-catenin #1	1.580x10^{-7} (Fig. 2A)	0.883 (Fig. 2A)
	siCtrl. vs. siβ-catenin #2	1.069x10^{-5} (Fig. 2A)	0.935 (Fig. 2A)
OE-33	siCtrl. vs. siβ-catenin #1	4.869x10^{-5} (Fig. 2B)	0.196 (Fig. 2B)
	siCtrl. vs. siβ-catenin #2	0.013 (Fig. 2B)	0.134 (Fig. 2B)
OAC-P4C	siCtrl. vs. siβ-catenin #1	0.341 (Fig. 2C)	0.499 (Fig. 2C)
	siCtrl. vs. siβ-catenin #2	0.137 (Fig. 2C)	0.175 (Fig. 2C)
FLO-1	siCtrl. vs. siβ-catenin #1	0.159 (Fig. 2D)	0.790 (Fig. 2D)
	siCtrl. vs. siβ-catenin #2	0.709 (Fig. 2D)	0.372 (Fig. 2D)
Kyse-150	siCtrl. vs. siβ-catenin #1	0.010 (Fig. 2E)	0.065 (Fig. 2E)
	siCtrl. vs. siβ-catenin #2	0.430 (Fig. 2E)	0.089 (Fig. 2E)
Kyse-180	siCtrl. vs. siβ-catenin #1	0.585 (Fig. 2F)	0.576 (Fig. 2F)
	siCtrl. vs. siβ-catenin #2	0.264 (Fig. 2F)	0.004 (Fig. 2F)
OAC-P4C	siCtrl. vs. siβ-catenin #1	0.213 (Fig. 3A)	n.a.
	siCtrl. vs. siβ-catenin #2	0.032 (Fig. 3A)	n.a.
FLO-1	siCtrl. vs. siβ-catenin #1	0.813 (Fig. 3B)	n.a.
	siCtrl. vs. siβ-catenin #2	0.792 (Fig. 3B)	n.a.
Kyse-150	siCtrl. vs. siβ-catenin #1	0.019 (Fig. 3C)	n.a.
	siCtrl. vs. siβ-catenin #2	0.044 (Fig. 3C)	n.a.
Kyse-180	siCtrl. vs. siβ-catenin #1	0.1158 (Fig. 3D)	n.a.
	siCtrl. vs. siβ-catenin #2	0.1196 (Fig. 3D)	n.a.
OE-19	Ctrl. vs. 2.5 μM XAV-939	1.564x10^{-8} (Fig. 4A)	1.531x10^{-4} (Fig. 4A)
	Ctrl. vs. 5 μM XAV-939	1.161x10^{-7} (Fig. 4A)	3.049x10^{-4} (Fig. 4A)
OE-33	Ctrl. vs. 5 μM XAV-939	0.004 (Fig. 4B)	0.140 (Fig. 4B)
	Ctrl. vs. 10 μM XAV-939	0.046 (Fig. 4B)	0.032 (Fig. 4B)
OAC-P4C	Ctrl. vs. 2.5 μM XAV-939	0.862 (Fig. 4C)	3.305x10^{-4} (Fig. 4C)
	Ctrl. vs. 5 μM XAV-939	0.690 (Fig. 4C)	1.626x10^{-4} (Fig. 4C)
FLO-1	Ctrl. vs. 2.5 μM XAV-939	0.981 (Fig. 4D)	4.609x10^{-7} (Fig. 4D)
	Ctrl. vs. 5 μM XAV-939	0.779 (Fig. 4D)	8.391x10^{-7} (Fig. 4D)
Kyse-180	Ctrl. vs. 2.5 μM XAV-939	0.087 (Fig. 4E)	0.029 (Fig. 4E)
	Ctrl. vs. 5 μM XAV-939	0.221 (Fig. 4E)	0.012 (Fig. 4E)

OE-19	Ctrl. vs. 5 μM JW55	0.024 (Fig. 5A)	$4.483x10^{-4}$ (Fig. 5A)
	Ctrl. vs. 10 μM JW55	0.017 (Fig. 5A)	$2.648x10^{-3}$ (Fig. 5A)
OE-33	Ctrl. vs. 5 μM JW55	0.098 (Fig. 5B)	0.185 (Fig. 5B)
	Ctrl. vs. 10 μM JW55	0.001 (Fig. 5B)	0.031 (Fig. 5B)
FLO-1	Ctrl. vs. 5 μM JW55	0.998 (Fig. 5C)	0.330 (Fig. 5C)
	Ctrl. vs. 10 μM JW55	0.805 (Fig. 5C)	0.006 (Fig. 5C)
Kyse-150	Ctrl. vs. 5 μM JW55	0.674 (Fig. 5D)	0.840 (Fig. 5D)
	Ctrl. vs. 10 μM JW55	0.411 (Fig. 5D)	0.486 (Fig. 5D)
Kyse-180	Ctrl. vs. 5 μM JW55	0.504 (Fig. 5E)	0.011 (Fig. 5E)
	Ctrl. vs. 10 μM JW55	0.971 (Fig. 5E)	0.026 (Fig. 5E)

[1] two-way analysis of variance (ANOVA), [2] unpaired two-sample Students t-test, RT = radiotherapy, CRT = chemoradiotherapy, CTB = CellTiter-Blue® Cell Viability Assay, FI = fractionated irradiation, n.a. = not applicable.

Table S2. Antibodies for Western blot analysis.

Protein	clone	Host	Size (kD)	Dilution	Incubation time	Company	Catalogue number
Actin	Polyclonal	Rabbit	42	1 : 10,000	Over night	Sigma-Aldrich	A2066
Axin2	Monoclonal	Rabbit	95, 98	1 : 1000	Over night	Cell Signaling	2151
Active β-catenin (ABC)	Monoclonal	Mouse	92	1 : 4000	Over night	Merck Millipore	05-665
Total β-catenin (TBC)	Polyclonal	Rabbit	92	1 : 2000	Over night	Cell Signaling	9587
TCF7L2	Monoclonal	Rabbit	68	1 : 10,000	Over night	Abcam	ab76151
Anti-rabbit IgG-HRP conjugated	n.a.	Goat	n.a.	1 : 30,000	2 h	Acris	R1364HRP
Anti-mouse IgG-HRP conjugated	n.a.	rabbit	n.a.	1 : 30,000	2 h	Acris	R1253HRP

kD = kilo Dalton, n.a. = not applicable, IgG = immunoglobulin G, HRP = horseradish peroxidase.

Table S3. Experimental conditions for dual luciferase reporter assays and cellular viability assays.

		DLR			CTB		
Cell line	Treatment	Cell number	Transfection reagent	DNA amount	Cell number	Transfection reagent	siRNA / lipid
OE-19	SuperFOP*Flas*h	100,000	X-tremeGENE HP	250 ng	n.a.	n.a.	n.a.
	SuperTOP*Flash*	100,000	X-tremeGENE HP	250 ng	n.a.	n.a.	n.a.
	β-catenin-S33Y	100,000	X-tremeGENE HP	250 ng	n.a.	n.a.	n.a.
	Renilla	100,000	X-tremeGENE HP	12.5 ng	n.a.	n.a.	n.a.
	5-FU	n.a.	n.a.	n.a.	2000	n.a.	n.a.
	siRNA	n.a.	n.a.	n.a.	2000	RNAiMAX	10 nM / 0.5 µl
	DMSO / XAV-939	n.a.	n.a.	n.a.	2000	n.a.	n.a.
	DMSO / JW55	n.a.	n.a.	n.a.	2000	n.a.	n.a.
OE-33	SuperFOP*Flas*h	150,000	X-tremeGENE HP	500 ng	n.a.	n.a.	n.a.
	SuperTOP*Flash*	150,000	X-tremeGENE HP	500 ng	n.a.	n.a.	n.a.
	β-catenin-S33Y	150,000	X-tremeGENE HP	500 ng	n.a.	n.a.	n.a.

	Renilla	150,000	X-tremeGENE HP	25 ng	n.a.	n.a.	n.a.
	5-FU	n.a.	n.a.	n.a.	2000	n.a.	n.a.
	siRNA	n.a.	n.a.	n.a.	2000	RNAiMAX	10 nM / 0.1 µl
	DMSO / XAV-939	n.a.	n.a.	n.a.	2000	n.a.	n.a.
	DMSO / JW55	n.a.	n.a.	n.a.	2000	n.a.	n.a.
OAC-P4C	SuperFOP*Flas*h	100,000	X-tremeGENE HP	1,000 ng	n.a.	n.a.	n.a.
	SuperTOP*Flash*	100,000	X-tremeGENE HP	1,000 ng	n.a.	n.a.	n.a.
	β-catenin-S33Y	100,000	X-tremeGENE HP	1,000 ng	n.a.	n.a.	n.a.
	Renilla	100,000	X-tremeGENE HP	50 ng	n.a.	n.a.	n.a.
	5-FU	n.a.	n.a.	n.a.	3000	n.a.	n.a.
	siRNA	n.a.	n.a.	n.a.	3000	RNAiMAX	10 nM / 0.3 µl
	DMSO / XAV-939	n.a.	n.a.	n.a.	3000	n.a.	n.a.
SK-GT4	5-FU	n.a.	n.a.	n.a.	3000	n.a.	n.a.
FLO-1	SuperFOP*Flas*h	100,000	X-tremeGENE HP	1000 ng	n.a.	n.a.	n.a.
	SuperTOP*Flash*	100,000	X-tremeGENE HP	1000 ng	n.a.	n.a.	n.a.
	β-catenin-S33Y	100,000	X-tremeGENE HP	1000 ng	n.a.	n.a.	n.a.
	Renilla	100,000	X-tremeGENE HP	50 ng	n.a.	n.a.	n.a.
	5-FU	n.a.	n.a.	n.a.	3000	n.a.	n.a.
	siRNA	n.a.	n.a.	n.a.	3000	RNAiMAX	10 nM / 0.1 µl
	DMSO / XAV-939	n.a.	n.a.	n.a.	3000	n.a.	n.a.
	DMSO / JW55	n.a.	n.a.	n.a.	3000	n.a.	n.a.
Kyse-70	5-FU	n.a.	n.a.	n.a.	2000	n.a.	n.a.
Kyse-150	SuperFOP*Flas*h	150,000	X-tremeGENE HP	1000 ng	n.a.	n.a.	n.a.
	SuperTOP*Flash*	150,000	X-tremeGENE HP	1000 ng	n.a.	n.a.	n.a.
	β-catenin-S33Y	150,000	X-tremeGENE HP	1000 ng	n.a.	n.a.	n.a.
	Renilla	150,000	X-tremeGENE HP	50 ng	n.a.	n.a.	n.a.
	5-FU	n.a.	n.a.	n.a.	2000	n.a.	n.a.
	siRNA	n.a.	n.a.	n.a.	2000	RNAiMAX	10 nM / 0.1 µl
	DMSO / JW55	n.a.	n.a.	n.a.	2000	n.a.	n.a.
Kyse-180	SuperFOP*Flas*h	100,000	X-tremeGENE HP	250 ng	n.a.	n.a.	n.a.
	SuperTOP*Flash*	100,000	X-tremeGENE HP	250 ng	n.a.	n.a.	n.a.
	β-catenin-S33Y	100,000	X-tremeGENE HP	250 ng	n.a.	n.a.	n.a.
	Renilla	100,000	X-tremeGENE HP	12.5ng	n.a.	n.a.	n.a.
	5-FU	n.a.	n.a.	n.a.	3000	n.a.	n.a.
	siRNA	n.a.	n.a.	n.a.	3000	RNAiMAX	10 nM / 0.1 µl
	DMSO / XAV-939	n.a.	n.a.	n.a.	3000	n.a.	n.a.
	DMSO / JW55	n.a.	n.a.	n.a.	3000	n.a.	n.a.
Kyse-270	5-FU	n.a.	n.a.	n.a.	2000	n.a.	n.a.

DLR = dual luciferase assay, CTB = CellTiter-Blue® Cell Viability Assay, 5-FU = 5-fluorouracil, n.a. = not applicable, S33Y = mutated β-catenin-S33Y.

Table S4. siRNA sequences.

Gene	Target sequence	Size (bp)	Accession number	Company	Catalogue number
Negative control (AllStarsNEG)	CAGGGTATCGACGATTACAAA	21	n.a.	Qiagen	SI03650318
β-catenin #1	CTCGGGATGTTCACAACCGAA	21	NM_001098209	Qiagen	SI02662478
β-catenin #2[1] (pool)	CAUCCUAGCUAUCGUUCUU	19	XM_006712983	Dharmacon	J-003482-09
	GGUACGAGCUGCUAUGUUC	19	NM_001098209	Dharmacon	J-003482-12

[1]siRNA β-catenin #2 consists of 2 siRNAs (#09 and #12) that were pooled, bp = base pair, n.a. = not applicable.

Table S5. Experimental conditions for colony formation assays.

Cell line	Treatment	Transfection method time pre-irradiation	Cell number 0, 1, 2 Gy	Cell number 4 Gy	Cell number 6 Gy	Cell number 8 Gy	Growth period (days)
OE-19	siRNA (16 nM)	Amaxa, 48 hours	500	1000	1500	2000	11
	DMSO / XAV-939	96 hours	500	1000	1500	2000	11
	DMSO / JW55	96 hours	500	1000	1500	2000	11
OE-33	siRNA (16 nM)	Amaxa, 24 hours	750	1500	2250	3000	7
	DMSO / XAV-939	24 hours	750	1500	2250	3000	7
	DMSO / JW55	24 hours	750	1500	2250	3000	7
OAC-P4C	siRNA (16 nM)	Amaxa, 72 hours	500	500	1000	1000	9
	DMSO / XAV-939	144 hours	500	500	1000	1000	9
SK-GT4	n.a.	n.a.	500	1000	1500	2000	7
FLO-1	siRNA (16 nM)	Amaxa, 72 hours	500	500	1000	1500	7
	DMSO / XAV-939	144 hours	500	500	1000	1500	7
	DMSO / JW55	48 hours	500	500	1000	1500	7
Kyse-70	n.a.	n.a.	250	500	750	1000	7
Kyse-150	siRNA (16 nM)	Amaxa, 24 hours	250	250	250	250	7
	DMSO / JW55	24 hours	250	250	250	250	7
Kyse-180	siRNA (16 nM)	Amaxa, 24 hours	200	400	600	800	7
	DMSO / XAV-939	144 hours	200	400	600	800	7
	DMSO / JW55	24 hours	200	400	600	800	7

CFA = colony formation assay, DMSO = dimethyl sulfoxide, n.a. = not applicable.
